# The Role of Host Species in Experimental Ferlavirus Infection: Comparison of a Single Strain in Ball Pythons (*Python regius*) and Corn Snakes (*Pantherophis guttatus*)

**DOI:** 10.3390/ani13172714

**Published:** 2023-08-26

**Authors:** Michael Pees, Annkatrin Möller, Volker Schmidt, Wieland Schroedl, Rachel E. Marschang

**Affiliations:** 1 Department of Small Mammal, Reptile and Avian Medicine, University of Veterinary Medicine Hannover, 30559 Hanover, Germany; 2 Tierarztpraxis Dr. Kühnel, 98527 Suhl, Germany; annkatrin_moeller@web.de; 3 Clinic for Birds and Reptiles, Veterinary Teaching Hospital, University of Leipzig, 04103 Leipzig, Germany; volker.schmidt@vogelklinik.uni-leipzig.de; 4 Institute of Bacteriology and Mycology, University of Leipzig, 04103 Leipzig, Germany; 5 Laboklin GmbH & Co. KG, 97688 Bad Kissingen, Germany; rachel.marschang@googlemail.com

**Keywords:** ball python, ferlavirus, infection, pathology, detection, PCR, cell culture, immunology, IgY, IgM

## Abstract

**Simple Summary:**

Paramyxoviruses in the genus *Ferlavirus* are well-documented pathogens in snakes. Disease severity appears to depend on multiple factors which are not fully understood. In order to further understand the role of host species in ferlaviral infection and disease, a genogroup B ferlavirus that had previously been shown to be highly pathogenic in corn snakes (*Pantherophis gutattus*) was inoculated into ball pythons (*Python regius*). The pythons became infected but developed much milder disease than that observed in the corn snakes. The corn snakes also had a higher rate of bacterial involvement in the lungs as well as much weaker humoral immune responses to infection. In both species, the respiratory tract was the primary target of the virus, but systemic spread was also observed. While this study supports previous findings indicating a wide host range among squamate reptiles for ferlaviruses, it also shows that specific host species can react very differently to infection with individual virus strains.

**Abstract:**

Ferlaviruses are a cause of respiratory disease in snakes. Four genogroups (A, B, C, and tortoise) have been described. Disease development is believed to depend on virus, host, and environment-specific factors. There is evidence of transmission of individual strains between genera and families of reptiles. A genogroup B virus previously used in a transmission study with corn snakes (*Pantherophis guttatus*) was applied intratracheally in ball pythons (*Python regius*) using the same protocol as for the corn snakes. Ball pythons became infected, with initial mild clinical signs noted four days post infection (p.i.), and the virus was detected first in the lungs on day 4 and spread to the intestine, pancreas, kidney and brain. Hematology showed an increase in circulating lymphocytes which peaked on day 28 p.i. Antibodies were detected beginning on day 16 and increased steadily to the end of the study. In comparison to corn snakes, ball pythons exhibited milder clinical signs and pathological changes, faster development of and higher antibody titers, and a hematological reaction dominated by lymphocytosis in contrast to heterophilia in corn snakes. These differences in host reaction to infection are important to understand ferlavirus epidemiology as well as for clinical medicine and diagnostic testing.

## 1. Introduction

Paramyxoviruses in the genus *Ferlavirus* were first described in snakes in the 1970s [1]. They have since been detected in a wide range of snake species as well as in lizards and, occasionally, in chelonians [2]. They are known to primarily cause respiratory disease in infected animals but have also been associated with central nervous system signs as well as systemic disease. The severity of clinical signs can range from inapparent or mild to severe with multiple reports of severe disease outbreaks in larger collections [1,3,4].

Characterization of several ferlavirus isolates based on partial genome sequences has shown that they can be divided into at least four different genogroups, referred to as A, B, C, and tortoise [5,6]. Studies have shown some serological differences between different strains [2,7]. Viruses in genogroups A and B appear to strongly cross-react, while viruses in genogroup C cross-react less strongly with A and B viruses [8]. Differences in host range and pathogenicity between the different genogroups and different strains are not yet understood [9], although various genotypes have been found in a variety of host species [10,11].

The first transmission study with ferlaviruses was conducted using intratracheal inoculation in a group of Aruba Island rattlesnakes (*Crotalus unicolor*) [12]. The virus used in that study was not further characterized. Five animals were inoculated and were euthanized or died between 4 and 22 days post infection (p.i.). Examination of lung tissue showed progressive pathological changes in the lungs of infected animals beginning 4 days p.i. The two animals that were not euthanized earlier died suddenly on days 19 and 22, respectively, both with blood found in their oral cavity. None of the snakes in that study developed antibodies against the virus used for inoculation as determined by hemagglutination inhibition assay (HI).

A transmission study using genogroup A, B, and C ferlaviruses in different groups of corn snakes (*Pantherophis guttatus*) showed distinct differences in pathogenicity between the viruses [9]. In that study, 12 animals in each group were inoculated intratracheally with a ferlavirus genogroup A, B, or C isolates and 3 animals were each euthanized and examined 4, 16, 28, and 49 days p.i. Significant differences were found in clinical disease and pathological changes between the study groups, with the most severe disease found in animals inoculated with the genogroup B virus. In this group, snakes developed severe clinical disease starting from day 16 p.i., and the last animals remaining had to be euthanized earlier than planned on day 35 p.i. due to disease. In contrast, only two animals infected with the group A virus developed moderate clinical signs, none died, and none had to be euthanized earlier than planned. In the group inoculated with the group C virus, two developed mild clinical signs, three died, and one had to be euthanized due to disease. Analysis of the immune responses of snakes to infection using HI, serum IgM and IgY concentrations, and hematology showed a leukocytosis that was detectable beginning 16 days p.i. and antibodies detectable beginning 28 days p.i. [8]. However, there were significant differences in immune response depending on the virus used. Animals that were infected with the genogroup B isolate developed the highest white blood cell counts (WBC), but antibodies were only detectable in a single animal at a low titer shortly before death (which in this group occurred on day 35). Animals in the groups that received the group A and C viruses developed high antibody titers (by HI) over the course of the study and showed significantly fewer clinical signs and pathological findings.

Differences in clinical signs and pathological findings between different reports of ferlavirus infections and outbreaks as well as in the transmission studies available indicate that a variety of factors including host-specific factors (e.g., species, age, health status) and virus-specific factors (e.g., genogroup and strain), as well as additional factors including temperature, other environmental factors, and secondary infections, likely play a role in infection and disease development. The aim of the current study was to evaluate the effects of host species on infection and disease using a genogroup B virus previously found to be highly pathogenic in corn snakes [9]. The same virus and protocol used in those experiments was used to infect ball pythons, and clinical, pathological and immunological reactions to infection were compared between the ball pythons and the previous results from the corn snakes.

## 2. Materials and Methods

The overall experimental design was identical to a study conducted with three different virus strains in corn snakes [8,9,13], as it was the aim to allow direct comparison of the study results. The animal trial was approved by the national authority (Landesdirektion Sachsen, application number TVV 61/13).

The study was conducted with 12 adult ball pythons (*Python regius*). The animals were captive-bred and acquired as adult healthy snakes from a commercial company. Nine of the snakes were female, three were male. Average body length (snout–cloaca) was 125 cm (range 92 cm to 161 cm), and mean body mass was 1409 g (range 644 g to 2977 g). No further details on individual histories of the snakes were available.

All animals underwent a thorough clinical examination following established standards. They were checked for endo- and ectoparasites and samples were taken (swabs from the choana and the cloaca, tracheal washes) and checked for aerobic bacterial and fungal growth as described previously [13]. A combined sample (tracheal wash and cloacal swab) was examined for the presence of ferlaviruses by PCR following an established protocol [14]. Briefly, the PCR targeted a 566 bp portion of the L-gene using primers F5-outer and R6-outer followed by F7-inner and R8-inner in a nested format as described by Ahne et al. [14] using the specific methods described by Kolesnik et al. [15]. This PCR has been shown to be highly specific and able to detect between 5 × 10^−1^ and 5 × 10^−3^ ng/µL viral RNA [15]. RNA prepared from a ferlavirus cell culture isolate was used as a positive control and HPLC water was used as a negative control. Only snakes without any remarkable result (clinically healthy, good body condition, negative ferlavirus PCR, negative for parasites, and bacterial and fungal flora assessed to be physiological) were included in the study. The snakes were housed for at least two months before the beginning of the study period, and examinations were repeated on day −6 (before virus inoculation), at the start of the study protocol.

The snakes were kept in two terraria of approximately 140 × 78 × 65 cm, with six snakes in each group. Husbandry conditions followed general recommendations for the species, including suitable ground material (turf), a water basin and hiding places. Data loggers (microlite II, imec, Heilbronn, Germany) were used to monitor temperature and humidity. Temperature range was 20 °C to 28 °C with a 14/10 h day/night rhythm and hot spots of 35 °C, relative humidity was kept in a range of 50% to 70%. During the complete study period, snakes were fed (one mouse per snake) every seven to ten days in separate boxes.

For experimental infections, the virus strain that proved to be most virulent in corn snakes in the preceding study [13] was chosen. This strain was isolated from a timber rattlesnake (*Crotalus horridus*) during a disease outbreak in a collection of various viper species in Germany [5]. The virus was cultured on viper heart cells (VH2) according to an established protocol [6], the virus suspension used was an aliquote of the same virus passage as that used in the corn snake study [13] that had been stored frozen at −80 °C. Using a tracheal tube, 1 mL of the prepared virus suspension was inoculated into the trachea, and the virus suspension was titrated on VH2 cells [13] and confirmed to contain 10^6.5^ TCID_50_ per mL.

No negative control group was used for this study, as the previous study with corn snakes included a group inoculated with a cell culture suspension without the virus. Since no clinical effect of this treatment was noted in the corn snakes, a similar treatment was considered unnecessary in the present study. Instead, the aim was to compare the results of both infection groups (corn snakes vs. ball pythons) directly.

The study and sampling design included a 6-day pre-inoculation period followed by the virus application and a 49-day sampling/experimental period. Over the whole study period, animals were checked daily by thorough inspection and weekly with a complete clinical examination including body mass determination. Clinical disease conditions for euthanasia were defined in the animal trial protocol as ongoing severe clinical signs including central nervous signs, severe respiratory distress leading to continuous dyspnea, or continuous signs of pain or apathy.

Samples were collected on days −6, 4, 16, 28 and 49, according to the protocol used for the corn snake study [13]. Tracheal washes and cloacal swabs and blood were collected intra vitam from all remaining snakes on each sampling day, including from those snakes that were euthanized after swabbing. On each of the days post infection (4, 16, 28 and 49), three snakes were selected randomly, euthanized, and complete necropsies were performed.

The post-mortem examination was conducted according to published standards [16]. All organs were assessed macroscopically, and the following tissues were processed for histopathological examination: lung, liver, kidney, small and large intestine, pancreas, spleen, brain, and gonads. Virus detection by PCR and virus isolation in cell culture was carried out using samples from the lungs, intestine, pancreas, kidney, and brain (see below). For details on sample collection protocols, see Pees et al. [13] (virus detection), Neul et al. [8] (immunology) and Pees et al. [9] (pathology). Lung tissue was also examined for bacteria and fungi according to Pees et al. [9].

Hematologic examination included a leukocyte count (estimation method according to Campbell and Ellis [17]) and differential blood cell counts (100 cells at 1000× magnification), which were calculated as absolute values. HI was performed as described previously [8,18] using the virus isolate used to inoculate the pythons. Plasma IgM and IgY levels were determined using indirect ELISA as described previously in corn snakes [8], also using the same virus isolate used to infect the animals. Briefly, concentrated, purified viral antigen was adsorbed onto microtiter plates. Bovine casein was used as a control. For IgM detection, plasma samples were diluted 1:200 in assay dilution buffer. A positive sample (pooled blood sample from the group) and a negative control sample (plasma sample from a confirmed ferlavirus-negative ball python) were added accordingly. Python IgM detection was carried out using 100 µL of horseradish peroxidase-conjugated rabbit-anti-IgM (isolated from corn snakes and carpet pythons)-IgGq diluted 1:10,000 in assay dilution buffer. For IgY detection, python plasma samples were diluted 1:100, and horseradish peroxidase-conjugated IgG (from rabbit)-anti-IgY (isolated from corn snakes and carpet pythons) was used. Optical densities (ODs) were used as relative values for assessment of the immunoglobulin production.

The virus was detected by PCR and virus isolation in cell culture as described previously [13] in tissue samples from euthanized animals on days 4, 16, 28, and 49. As in the study in corn snakes, after initial sampling on day −6, intra vitam samples were tested beginning on day 16. No intra vitam samples were tested for ferlaviruses on day 4 following the protocol from the corn snake study, which was designed to rule out any chance of false positive PCR results due to the persistence of viral RNA at the inoculation site.

For PCR detection, samples were cooled to 4 °C in a microtube container without preservatives and sent overnight to a diagnostic laboratory (Laboklin GmbH & Co. KG, Bad Kissingen, Germany). Further processing was carried out according to established protocols [14] and the previous study design [13], using primers targeting a portion of the ferlaviral L gene as described above.

For virus isolation in cell culture, samples were placed in microtube containers, and for intra vitam cloacal and tracheal wash samples, a transport medium was added (Remel micro test M4, Remel, Dartford, UK). Samples were immediately frozen at −80 °C. Isolation was carried out using VH2 as described [19]. Samples were thawed and prepared as described in detail elsewhere [13]. Both VH2 subculture suspensions, as well as VH2 monolayers (each in 96-well plates), were used for virus isolation attempts. Cells were incubated at 28 °C and checked daily for cytopathic effects (CPE) or cell toxicity for eight days, and up to two passages were prepared. Samples in which no CPE was observed throughout the two passages were declared negative, whereas culture samples with CPE and positive PCR confirmation were declared positive.

Statistical analysis was performed using the software SPSS 22.0 (IBM, Armonk, NY, USA). As the majority of the values were not normally distributed, all values are reported as medians and interquartile ranges (25th to 75th percentile). For comparisons between sampling days, the Friedman test for multiple samples was used, followed by the Wilcoxon signed rank test in cases in which significance was determined (hematology and immunology). A *p* value of 0.05 or less was considered significant.

## 3. Results

### 3.1. Clinical and Post-Mortem Findings

Overviews comparing clinical and post-mortem findings in ball pythons and corn snakes are provided in Table 1, Table 2 and Table 3. At the beginning of the ball python study, all snakes were clinically unremarkable. Following inoculation of the virus, moderate clinical signs were first observed on day 4 (Table 1). On day 16, several snakes demonstrated reddening or mild signs of stomatitis in the oral cavity, or the tracheal wash sample was flaky. Similar signs (mucous secretion, stomatitis, or flaky tracheal wash sample) were observed in a majority of the snakes up to day 49. Severe clinical signs such as respiratory sounds were found only intermittently over the course of the study. No snake died spontaneously or had to be euthanized during the study due to disease conditions.

**Figure 1:**
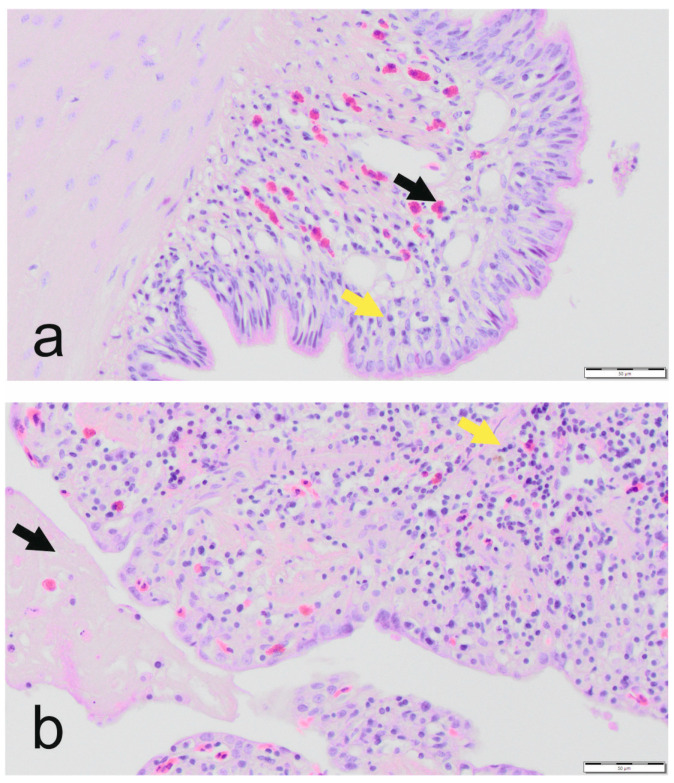
Histologic lung assessment: examples for (**a**) mild, and (**b**) severe changes of the lung tissue of ball pythons (*Python regius*) following infection with a genotype B ferlavirus. Magnification 400×, H&E stain. (**a**) mild subepithelial lymphoplasmacytic (yellow arrow) and heterophilic infiltrates (black arrow); (**b**) severe diffuse heterophilic and lymphoplasmacytic infiltration in the interstitium (yellow arrow), fibrin in faveolar space (black arrow).

Histological changes in the lungs of infected ball pythons ranged from mild to moderate (Table 2, Figure 1), and none of the changes were judged to be severe as determined previously during the corn snake study (Figure 1).

Other histological findings in the ball pythons included intracytoplasmic brown and Prussian blue positive pigment in the hepatocytes (8/12) and in the renal tubule cells (12/12) and melanomacrophages in the liver (12/12). Mild multifocal lymphoplasmacytic proliferation was seen in the enteric submucosa (1/12), in the renal interstitium (1/12) and perivascular in the liver (1/12). One snake presented with multifocal pancreatic necrosis (1/12) and vacuolization of adrenal cortex cells was seen in four snakes (4/12). Hemosiderosis in the liver and hemosiderotic pigment nephrosis were also detected in corn snakes (5/12). Additionally, acute reactive splenohepatitis (6/12), acute catarrhal enteritis (1/12), epicarditis (1/12) and acute necrotizing pancreatitis (1/12) were diagnosed in corn snakes infected with the same virus isolate [9].

Microbiologically, no fungi could be isolated from the lung tissue. *Salmonella* spp. were isolated from the lungs of two ball pythons on day 49, and *Citrobacter freundii* was cultured from the lung of one snake on day 4 (Table 3).

### 3.2. Immune Reaction

#### 3.2.1. Hematology

**Figure 2:**
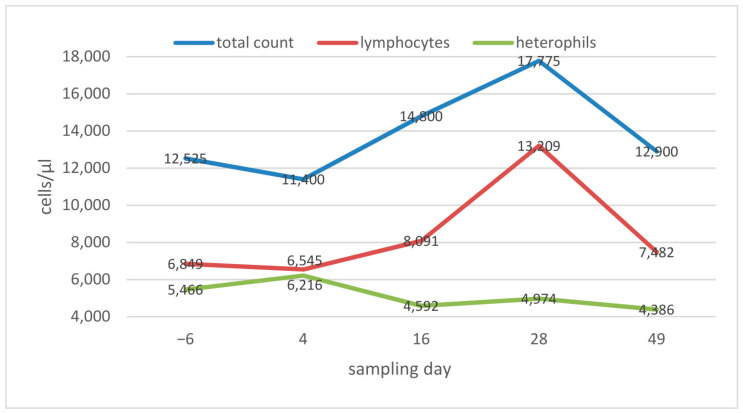
Haematology results in ball pythons (*Python regius*): white blood cell counts, calculated heterophil and lymphocyte counts, shown as median values per sampling day.

The baseline value for the total estimated white blood cell count was determined on day −6 with a median of 12,525 cells/µL, with a broad range of 7500 cells/µL to 25,400 cells/µL. Regarding the median, no increase was seen on day 4 post infection, but from day 16 to day 28, the number of white blood cells increased to a median of 17,755 cells/mL, before dropping again to almost base levels at the end of the study. Calculating the total number of heterophils and lymphocytes based on the differential blood cell count, it can be seen that this increase was mainly triggered by an increase in the number of lymphocytes (of almost 100% between day −6 and day 28), whereas the number of heterophils remained almost constant during the course of the study (Figure 2). Despite the clear increase in the median to day 28, the differences between the sampling days were neither significant for lymphocytes nor for heterophils.

**Figure 3:**
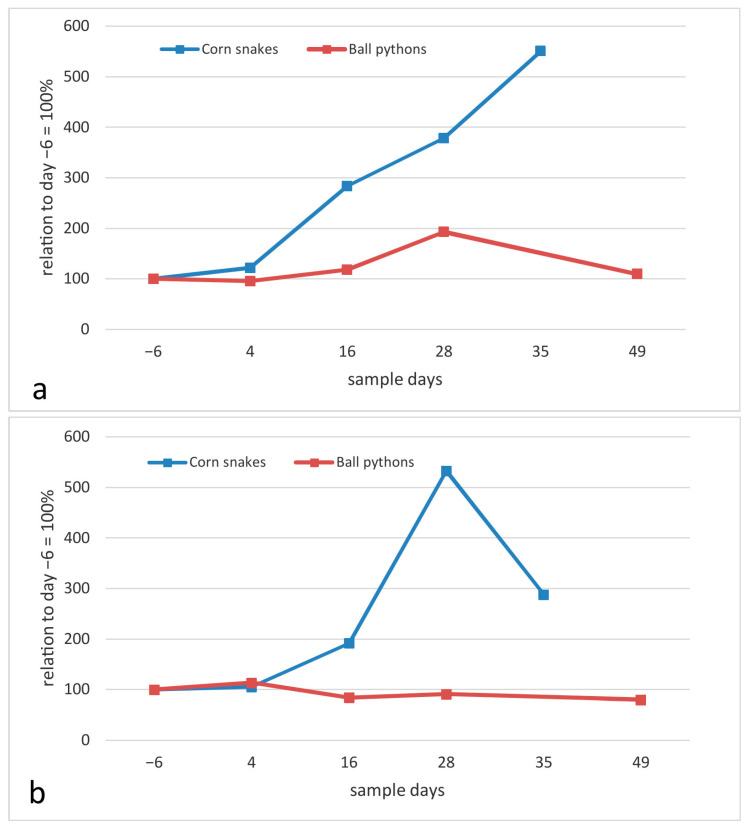
Blood leukocyte counts, relative changes (in percent, median values), (**a**) total lymphocyte count, (**b**) total heterophil count. Ball pythons (*Python regius*) in this study in comparison to results in corn snakes (*Pantherophis guttatus*) [8].

In comparison with the ball pythons, the relative increase in lymphocyte counts following infection was even higher for the corn snakes (Figure 3a, comparison to day −6), and continued until the last day of the study (day 35). In contrast to the ball pythons, the heterophil count also rose starkly in the corn snakes (Figure 3b).

#### 3.2.2. Hemagglutination Inhibition Assays (HI)

**Figure 4:**
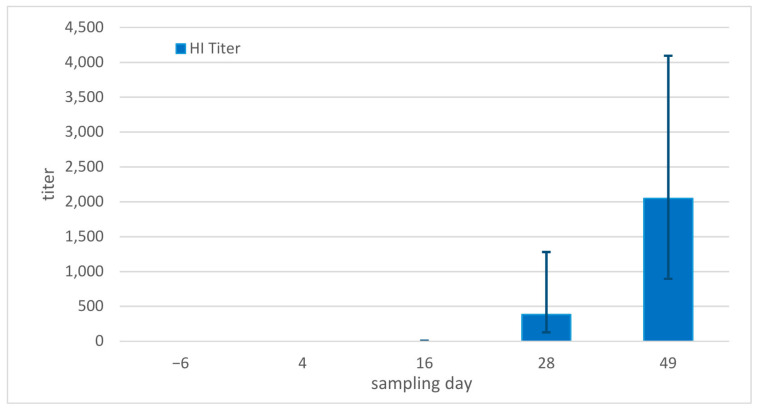
Hemagglutination inhibition assay (HI) titers in ball pythons (*Python regius*), median values and ranges per sampling day.

An HI titer of <2 was determined in all snakes on day −6. No antibodies were detected in any of the snakes on day 4 post infection either (all < 2). On day 16, HI titers of between 4 and 128 were detected in four snakes (4/9). On day 28, all remaining snakes were positive, with titers between 256 and 4096. On day 49, titers between 256 and 2048 were measured in the three remaining snakes (Figure 4). Statistical comparison confirmed a significant change over the sampling days (Friedman test, *p* = 0.022), and a significant increase in titers when comparing titers on days −6, 4, and 16 separately with results from day 28 (*p* = 0.026 to 0.027).

In comparison, in the corn snake study, only a single corn snake at the end of the study (on day 35) had detectable antibodies when the animal was euthanized due to clinical disease [8].

#### 3.2.3. IgM and IgY Determination

**Figure 5:**
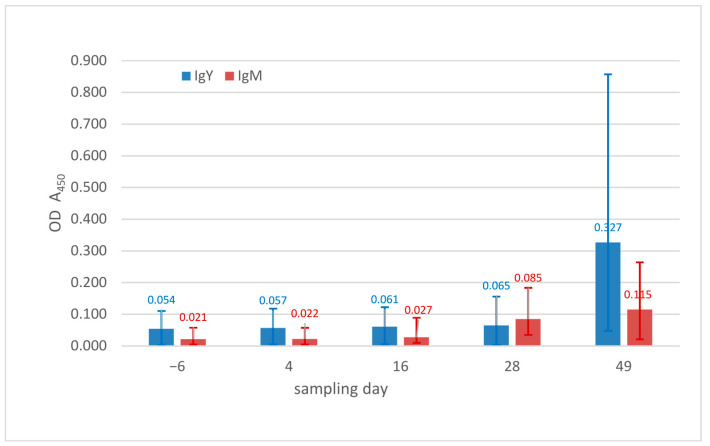
IgY and IgM concentrations in ball pythons (*Python regius*), optical density values, for each sampling day (median, 1st and 3rd quartile) (OD—optical density).

Baseline OD median values in the ball pythons were very similar on days −6 and day 4 p.i. On day 16, only a slight increase in the ODs was measured, with a more prominent increase in IgM levels than in IgY levels. On day 28, the IgM OD median had increased by 290% and on day 49, by 548% in comparison to day −6. For IgY, the median OD had only increased by 16% on day 28, but had increased by 494% on day 49, indicating a later production of IgY (Figure 5). Comparisons in antibody detection showed significant increases for IgY over the course of the sampling days (Friedman test, *p* = 0.032, for IgM *p* = 0.052), and significant increases in the IgY concentrations from day −6 to day 28 (*p* = 0.043) and from day 16 to day 28 (*p* = 0.028).

**Figure 6:**
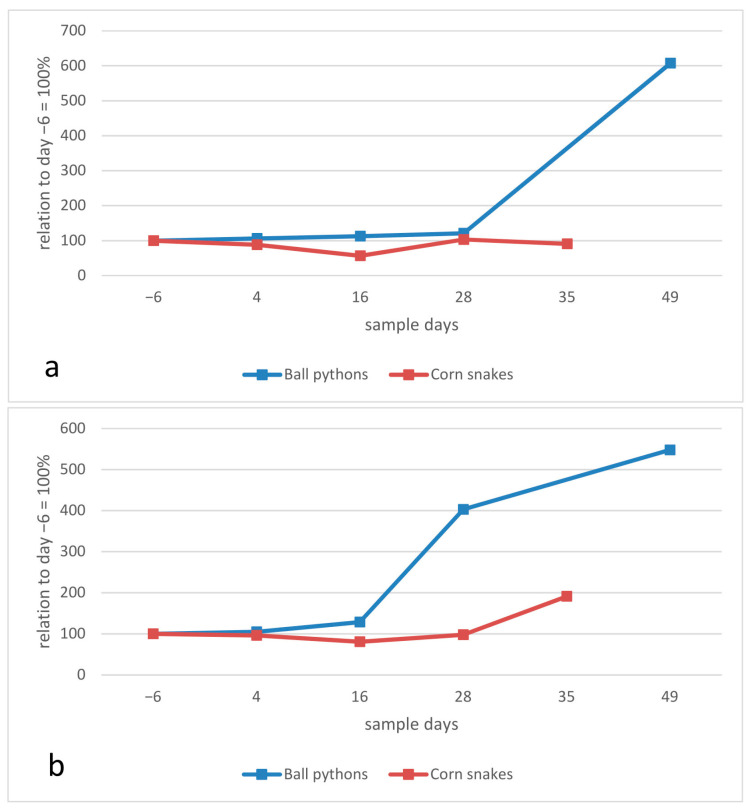
Relative changes in the measured optical densities reflecting concentration of IgM (**a**) and IgY (**b**) (median values) compared to day −6, ball pythons (*Python regius*) in this study in comparison to results in corn snakes (*Pantherophis guttatus*) [8].

A comparison of the relative changes in IgM and IgY levels in ball pythons and corn snakes over time showed no measurable increase in IgM levels and a much weaker IGY response to infection in corn snakes (Figure 6a,b).

### 3.3. Virus Detection

All tracheal wash samples examined were PCR positive, but the virus was only isolated in cell culture from 61% (11/18) (Table 4). PCR detected a ferlavirus in 72% (13/18) of the cloacal swabs tested, while virus isolation was successful in 61% (11/18). Regarding the sampling days, and assuming that one positive detection (PCR or cell culture) is sufficient to prove virus shedding, cloacal swabs were positive in 89% on day 16 (8/12), 67% on day 28 (4/6) and 100% on day 49 (3/3).

**Figure 7:**
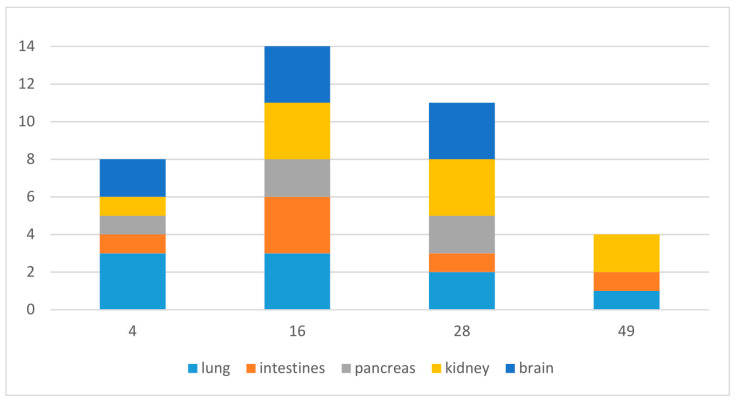
Ferlavirus-positive tissue samples per day (positive in PCR and/or cell culture), maximum would be 15 (three animals, five organs).

Of the tissue samples tested following necropsy (Table 5, Figure 7), 52% were PCR positive (31/60), and the virus was isolated in cell culture from 33% (20/60). In total, 43% of the samples that were tested negative in cell culture were positive in PCR (17/40), but only 21% (6/29) of the samples that were tested negative by PCR were positive in cell culture.

Virus detection by at least one of the methods used was successful in 75% of the lung samples, 50% of the intestinal samples, 42% of the pancreas samples, 75% of the kidney samples, and 67% of the brain samples. Regarding the sampling days, the percentage of positive detections in all organs together was 53% on day 4, 93% on day 16, 73% on day 28 and 27% on day 49 (Figure 7).

## 4. Discussion

This study aimed to compare the effects of an experimental ferlavirus infection in ball pythons (*Python regius*) directly to published results in corn snakes (*Pantherophis guttatus*). For the latter species defined infections with different strains were conducted and the consequences were evaluated and published in several manuscripts [8,9,13]. Previous work has shown that ferlaviruses are capable of infecting a wide range of reptile species [5,10,11,14,20,21,22], while some evidence has suggested that a number of factors may influence the course of clinical disease and pathological changes in infected animals. Ferlavirus infections have most often been documented in viperid species [1,3,4,23,24,25,26,27,28], often during severe disease outbreaks in collections. They have also been found in wild-caught viperids from South America [29]. Reports of infection in colubrids are slightly rarer, and include colubrid infections in mixed collections together with viperid snakes as well as in cases in which no contact with viperid snakes was reported [6,10,24,28,30,31]. There are a number of studies reporting detection of ferlaviruses in pythons. In some cases, animals were reported with clinical signs of disease, most often affecting the respiratory system [10,24,28] as well as the central nervous system [10,32], while studies screening boas and pythons in Germany for ferlaviruses by PCR reported no correlation between virus detection and disease [33,34]. While both host species and virus strain have been shown to play a role in disease development, reporting of virus characterization has been limited in many cases, making it difficult to determine what role the host species might play in disease development. The present study was designed to compare the effects of a single virus isolate found to be highly pathogenic in one species (corn snakes) on another host species (ball pythons).

In cases in which virus characterization has been reported, genogroup B viruses have been associated with respiratory or CNS disease or sudden death in boas, colubrids, pythons, and vipers in various collections in Europe [20]; with an outbreak of respiratory disease and high mortality in a mixed collection of elapids, vipers, and pythons in Croatia [35]; with colubrids or elapids experiencing respiratory disease in snake farms in China [31]; and with viperids, colubrids, elapids, and pythons with respiratory disease (but not in clinically healthy snakes) in several collections in Thailand [28].

### 4.1. Clinical and Post-Mortem Results

In the pythons, clinical signs were first detected in individual animals on day 4 post infection, and only mild to moderate clinical signs were detected in any of the infected animals over the course of the study (Table 1). This is in stark contrast to the results in corn snakes, where six snakes developed severe clinical signs and the last snake died on day 35 p.i. (Table 1). This difference in the reaction to the virus is also reflected in the pathological changes in the lung (Table 2). In corn snakes, in all snakes except those from sampling day 4, severe macroscopic alterations were found, and the histology results strongly corresponded with this; in the ball pythons, macroscopic changes in the lungs were only considered severe in a single animal in the study (on day 16). These differences in reaction are also seen in the histological findings, with findings in the ball pythons considered severe only in the single animal on day 16, and all other snakes considered either unremarkable or showing mild changes. These results demonstrate that the pathogenic effects of this virus isolate in these two species were very different, and the corn snakes appear to be much more susceptible to clinical disease caused by the virus strain used. This virus isolate was originally obtained from a timber rattlesnake during what was reported to be a severe disease outbreak in a collection of viperid snakes [5]. While it is possible that passages in cell culture or other factors in the laboratory could affect the pathogenicity of the virus, the same passage was used for both the corn snake study and the ball python study described here, indicating that host-specific factors were the most likely source for the differences in clinical course noted.

In addition to the overall clinical condition of the infected animals and the impact of infection on the lungs, indications of systemic disease were found in both species. Pancreas necrosis and lymphoplasmacytic proliferation in the small intestine, liver, and kidney were all noted in snakes in both studies. The virus was also detected in the affected tissues. It is, however, not clear if all of the changes observed were directly related to the ferlavirus infection.

Another factor that could have influenced the clinical and pathological findings found in each of these studies is secondary bacterial infections of the lung. In corn snakes, bacteria, including various *Salmonella* strains, were detected in the lungs of seven of the snakes examined (all from day 28 onwards). In contrast, bacteria were isolated from a much lower number of the infected ball pythons (three animals total). These detections might be incidental findings. However, the bacteria found—*Citrobacter freundii* and *Salmonella*—have been reported previously as causes of lung disease in snakes, also in combination with ferlavirus infections [9,33,34]. Altogether, these results also confirm a lower impact but a general susceptibility of ball pythons to ferlavirus infection.

### 4.2. Immune Reaction

Changes in white cell counts were one of the parameters used to help evaluate the general immune response to infection. The initial values were within published reference intervals [36]. However, the values of the individual snakes in the study already varied within a broad range. This variability in clinically healthy, uninfected animals, as well as limited sample size and the uncertainties inherent in the estimation method used to determine WBC, meant that despite a clear trend of increasing WBC, the changes over the course of the study were not found to be significant when comparing the sampling days.

The absolute and relative increase in the lymphocyte count (Figure 2 and Figure 3) indicates a strong lymphocyte reaction, which has been reported in some viral infections of reptiles [37,38], although no data are currently available on hematological evaluation of snakes naturally infected with ferlaviruses.

Within the limitations mentioned above, the change in hematological values in the individual ball pythons indicates a general immune response to the ferlavirus infection. The hematological reaction also appears to recede in the ball pythons with lower lymphocyte numbers measured on the final day of the study (day 49), which also corresponds to the histological findings. The differences in the lymphocyte and heterophil reactions in the corn snakes compared to the ball pythons could help explain the differences in the impact of the virus on each of these species. The massive heterophil reaction in corn snakes is a sign of an inflammatory response [38], possibly directly connected to secondary bacterial reactions. As discussed above, at the end of the study, bacteria were isolated from the lung tissues of all of the corn snakes, and corresponding histologic findings confirmed lung infection and inflammation. Increased numbers of heterophils and an increased heterophil:lymphocyte ratio have also been described in infections of turtles with ranaviruses, and the associated inflammation was hypothesized to have contributed to high mortality rates in that study [39].

The antibody reaction was measured according to Neul et al. [8] and focused on the standardized hemagglutination inhibition assay as well as the specific measurement of the IgM and IgY responses. The HI results show a strong antibody response in all pythons included in the study beginning on day 16, and increasing continuously to day 49. The ball pythons thereby showed a much stronger reaction, as antibodies were only detected in a single corn snake at the end of the study [8]. Interestingly, in corn snakes, the less virulent strains (genogroups A and C strains) both caused a stronger humeral immune reaction, with median titers up to 256, similar to the reaction observed against the genogroup B virus in the ball pythons. In the corn snake study, it was hypothesized that the lack of antibody production might be an important factor in the clinical course of disease and could have played a role in the severe disease caused by the genogroup B virus.

A comparison of the results of the IgM and IgY detection between the corn snake study [8] and the ball pythons showed similar dynamics to those seen for the HIs. In the ball pythons, the onset of the specific antibody production can clearly be seen from day 16 onwards, with an initial increase in IgM concentration, followed by IgY. IgM has been well documented as the initial antibody produced in response to infection in reptiles, while IgY is the long-term immunoglobulin [40]. The comparison to the values obtained for corn snakes again demonstrates the lack of an adequate adaptive immune response to this particular virus, while reactions to infection with other virus strains (of genogroups A and C) led to similar increases in both IgM and IgY to those seen against the group B virus in ball pythons, with similar timelines. Even with some limitations due to the small sample size on the last study days, the almost complete lack of a measurable antibody response in the corn snakes again demonstrates the inability of these animals to mount an adequate antibody response to this specific virus strain. As for the hematology and HI results, the comparison demonstrates a very different immune reaction in the two species, with a stronger inflammatory response in corn snakes and a stronger adaptive immune response in ball pythons. Since both species were infected with the same virus strain and aliquotes of the same virus preparation, it is most likely that these differences represent host-specific differences in reaction to infection. Why these reactions were so different remains unknown. While the immune parameters measured here do not represent the entirety of the possible immune response to infection, it is possible to hypothesize that a stronger adaptive immune response to infection in the ball pythons than in the corn snakes likely contributed to the less severe clinical disease and pathology found in the ball pythons, while inflammation likely contributed to disease development in the corn snakes.

### 4.3. Virus Detection

As previously reported for corn snakes, the PCR used in both studies was found to be more sensitive than virus isolation for ferlavirus detection in the ball pythons. All of the tracheal wash samples tested were PCR-positive beginning on day 16 to the end of the study. In contrast, while cell culture was also positive in all animals on day 16, almost all samples on days 28 and 49 were negative. Shedding of the virus appeared to differ between ball pythons and corn snakes. In both species, the virus was detectable by PCR in tracheal washes in most or all cases beginning on day 16. However, in the corn snakes, no virus was detectable in any of the cloacal swabs tested on day 16, and only in two out of three cloacal swabs tested on day 28 [13]. The PCR used in both studies was the same. More recent comparisons have indicated that the sensitivity of various PCRs can influence virus detection [15,31]. In a study comparing three PCRs for the detection of ferlaviruses, the PCR used in this study was found to be highly specific but less sensitive than a single-round PCR targeting a shorter segment of the same gene [15]. However, for this study, it was considered important to use the same methods as those used for the corn snake study [13] to allow direct comparison of results. Some studies have shown difficulties in detecting ferlaviruses in samples from live snakes, with negative PCR results reported from tracheal washes, while the virus was detectable in lung tissue [27]. In the present study, high infection loads may have led to better virus detection in tracheal washes than would be expected in naturally infected animals, in which time post infection, infectious dose, infection route, and immune status are all unknown.

The general virus prevalence in organs in the ball pythons (Figure 4) demonstrated an increase to day 16 with almost 100% positive samples in all organs, and then a reduction to 26% on day 49. Interestingly, this was similar in corn snakes with other virus strains, but with an identical virus, the detection rate increased continuously to 100% on the last study day (35) [13]. Together with the antibody and hematology results, this finding strengthens the hypothesis that ball pythons were better able to combat infection and therefore developed less severe disease than corn snakes infected with the same virus strain. Whether this would be true for other virus strains, or if host-specific response to infection is dependent on a combination of host species and virus strain, remains to be studied.

In both ball pythons and corn snakes, the ferlavirus was able to spread from the lungs to various other tissues, including all of the tissues tested (intestine, pancreas, kidney, and brain), although clinical signs were restricted to the respiratory tract. It is remarkable that virus detection in tissues other than the lung was possible earlier in ball pythons than in corn snakes, correlating with the higher rate of ferlavirus detection in cloacal swabs in the ball pythons. This more rapid spread throughout the body was not associated with higher pathogenicity. It appears that tissue distribution and shedding are not correlated with disease severity for this virus. However, it is possible that the more rapid spread of the virus to various tissues in the ball pythons could have contributed to the stronger adaptive immune response in this species. Receptor specificity has been studied and found to influence tissue tropism, pathogenicity, and immune response to paramyxoviruses in a wide variety of virus/host systems [41]. Systemic spread of virus has been correlated with specific motifs in the viral glycoproteins of many paramyxoviruses [40,42,43], and with higher pathogenicity in many cases. While sequence data from the genes encoding the viral surface glycoproteins F and HN have been determined for several ferlavirus isolates [44,45] including the one used in this study [9], the effects of specific changes or motifs in these receptor-binding proteins are not known, nor have the receptors in various tissues in reptiles been studied.

It is notable that while the lung samples were negative for virus detection in individual animals towards the end of the study, tracheal washes remained virus-positive in PCR tests. This is in contrast to previous reports in which virus detection in samples from tracheal washes was less sensitive than from lung samples in naturally infected vipers [27]. There are many possible reasons for this discrepancy including differences in time post infection, inoculation of a relatively high amount of virus in this transmission study, the ability of the PCR to detect RNA from viruses no longer capable of replicating, or a phase of infection in which virus has been cleared from the lung but not from the trachea.

### 4.4. Limitations of the Study Design

Due to the nature of experimental infections, this study has several limitations that need to be considered when interpreting the data. The study is focused on a defined group of only 12 animals, and for some sample days, the sample number was limited to three. On the other hand, the study design allowed for defined conditions and paired samples. The high virus load used in the study likely does not reflect the conditions during a natural infection. Ferlaviruses are likely spread by aerosol or direct contact between animals [1,2,46], but the amount of virus transmitted naturally is likely much lower than that used here. This study only used one defined virus strain, and no conclusions can be drawn regarding general differences between specific species with respect to ferlavirus susceptibility.

The lack of a control group of sham-inoculated ball pythons for direct comparison with the infected animals is a further limitation of the present study. The reason no control group was used was that, since the same protocol was used as in the previous study with corn snakes [13], it was deemed unlikely to provide any additional information, since the consequences of the application of cell culture supernatant (without virus) for a placebo group had already been described and discussed. It also conformed with the RRR principle (reduce refine replace) for animal trials.

## 5. Conclusions

The genogroup B ferlavirus strain used in this study was clearly able to infect and cause typical disease in the ball pythons. However, differences in severity of disease between corn snakes and ball pythons infected with the same strain demonstrate species-specific differences in virus pathogenicity as well as in host reaction to infection. These differences are relevant for clinical reptile medicine, virus epidemiology, and for diagnostic testing. Additional studies to understand the molecular basis of the differences should be carried out.

## Figures and Tables

**Table 1:** Changes in the respiratory tract of ball pythons (*Python regius*) and corn snakes (*Pantherophis guttatus*) [9] following infection with a genotype B ferlavirus. (n.t.—not tested, as animals died or were euthanized).

Days p.i.	Signs of Respiratory Disease	Number Affected/Observed	
		Ball Pythons	Corn Snakes [9]
1–4	Mucous secretion in the oral cavity	4/12	0/12
5–16	Flaky tracheal wash sample	4/9	1/9
	Reddening tracheal opening or mild signs of stomatitis	2/9	0/9
	Purulent secretion in the oral cavity	0/9	1/9
	Respiratory sounds	1/9	0/9
17–28	Mucous secretion in the oral cavity	3/6	0/6
	Flaky tracheal wash sample	3/6	4/6
	Respiratory sounds	2/6	0/6
	Purulent secretion in the oral cavity	0/6	1/6
	Acute death	0/6	2/6 (days 27, 28)
29–49	Mucous secretion in the oral cavity	3/3	0/3
	Flaky tracheal wash sample	3/3	n.t.
	Apathy, abnormal position	0/3	2/3
	Acute death	0/3	1/3 (day 33)
	Euthanasia due to clinical disease	0/3	2/3 (day 35)

**Table 2:** Gross and histological findings observed in the lungs of ball pythons (*Python regius*) and corn snakes (*Pantherophis guttatus*) [9] following infection with a genotype B ferlavirus. Finding are categorized as mild, moderate, or severe.

Day p.i.	Criteria	Findings	Number Affected	
			Ball Pythons	Corn Snakes [9]
4	Gross lesions	Mild colorless mucous	3/3	
	Histology	Mild multifocal subepithelial lymphoplasmacytic infiltrates	3/3	
16	Gross lesions	Mild colorless mucous	2/3	
		Moderate amount of colorless mucous combined with mild tissue thickening	1/3	
		Severe tissue thickening, reddish discoloration, yellow mucous		3/3
	Histology	Mild multifocal subepithelial lymphoplasmacytic infiltrates	1/3	
		Moderate diffuse heterophilic and lymphoplasmacytic interstitial infiltrates, edema	1/3	
		Severe diffuse heterophilic and lymphoplasmacytic infiltration in the interstitium, fibrin in faveolar space		3/3
28	Gross lesions	Mild colorless mucous	3/3	
		Severe tissue thickening, reddish discoloration, yellow mucous		3/3
	Histology	Mild multifocal subepithelial lymphoplasmacytic infiltrates	3/3	
		Severe diffuse heterophilic and lymphoplasmacytic infiltration in the interstitium, fibrin in faveolar space		3/3
49 (35)	Gross lesions	Mild colorless mucous	3/3	
		Severe tissue thickening, reddish discoloration, yellow mucous		3/3
	Histology	Mild subepithelial lymphoplasmacytic infiltrates	1/3	
		Severe diffuse heterophilic and lymphoplasmacytic infiltration in the interstitium, fibrin in faveolar space		3/3

**Table 3:** Bacteria isolated from the lungs of necropsied ball pythons (*Python regius*) and corn snakes (*Pantherophis guttatus*) [9] following infection with a genotype B ferlavirus.

Day p.i.	Ball Pythons		Corn Snakes	
	Number Affected	Bacteria Isolated	Number Affected	Bacteria Isolated
4	1/3	*Citrobacter freundii*	0/3	
16	0/3		1/3	*Salmonella* ssp. IIIa 41:z4,z23;-
28	0/3		3/3	2× *Salmonella* ssp. IIIb 48:l.v:1,5; *Citrobacter freundii*
49	2/3	1× *Salmonella* Treforest (1),51:z:1,6; 1× *Salmonella* Apeyeme 8,20:z38:-	3/3	*Salmonella* ssp. IIIb 14:z10:5; *Salmonella* ssp. IIIb 48:l,v:1,5; *Salmonella* Georgia 6,7:b:e:n:z15; 2× *Klebsiella pneumoniae*

**Table 4:** Results of PCR and cell culture isolation for ferlavirus detection in intra vitam samples from ball pythons (*Python regius*) for each sampling day (days −6, 16, 28, 49). Results are shown as PCR/cell culture.

No.	Tracheal Wash				Cloacal Swab			
	day −6	day 16	day 28	day 49	day −6	day 16	day 28	day 49
4	−/−	+/+			−/−	+/+		
5	−/−	+/+			−/−	−/+		
6	−/−	+/+			−/−	+/+		
7	−/−	+/+	+/−		−/−	+/+	+/+	
8	−/−	+/+	+/+		−/−	+/+	+/−	
9	−/−	+/+	+/−		−/−	−/+	−/−	
10	−/−	+/+	+/−	+/−	−/−	+/+	+/+	+/−
11	−/−	+/+	+/−	+/−	−/−	−/−	+/−	+/−
12	−/−	+/+	+/−	+/+	−/−	+/+	−/−	+/+

**Table 5:** Results of PCR and cell culture isolation for the detection of ferlaviruses in tissue samples from each ball python (*Python regius*) following euthanasia on day 4, 16, 28, or 49. Results are shown as PCR/cell culture.

Day	No.	Lung	Intestine	Pancreas	Kidney	Brain
day 4	1	+/+	−/−	−/−	−/−	−/−
	2	+/+	−/−	+/−	−/+	+/−
	3	+/+	+/+	−/−	−/−	−/+
day 16	4	+/+	−/+	−/+	+/+	+/−
	5	+/+	−/+	−/−	−/+	+/+
	6	+/+	+/−	+/−	+/−	+/−
day 28	7	+/+	−/−	+/+	+/−	+/−
	8	+/−	+/+	−/−	+/−	+/−
	9	−/−	−/−	+/−	+/−	+/−
day 49	10	−/−	−/−	−/−	+/−	−/−
	11	−/−	+/−	−/−	−/−	−/−
	12	+/+	−/−	−/−	+/+	−/−
all		9/8	4/4	4/2	7/4	7/2

## Data Availability

The data presented in this study are openly available in osf.io at https://doi.org/10.17605/OSF.IO/NRFW6 (accessed on 30 March 2023).
